# 8.F. Round table: Digital Innovations in Health Financing: Experiences from sub-Saharan Africa

**DOI:** 10.1093/eurpub/ckac129.496

**Published:** 2022-10-25

**Authors:** Organised by: DigitalUHC Consortium, Chair persons: Wilm Quentin (Germany), Catherine Kyobuutungi (Kenya)

## Abstract

**Background:**

Universal Health Coverage (UHC) is key for reaching Sustainable Development Goal 3. Digital technologies for health financing (DTHF) may help countries in sub-Saharan Africa (SSA) make progress towards achieving UHC. DTHF are increasingly used to strengthen health financing systems across their three key functions, (1) raising revenues, (2) pooling resources, and (3) purchasing services.

**Objectives:**

To provide an overview of the potential role of DTHF, discuss country case-studies of DTHF, explore possible risks and challenges, and outline a future research agenda. Together with the participants, we intend to discuss policy options to enable maximising the benefits, while mitigating the risks and challenges.

Added value: The workshop will (1) identify potential benefits as well as risks and challenges of DTHF; (2) highlight the importance of politics and policies for reaping the benefits and minimizing their risks; and (3) establish the building blocks of a future research agenda on digital health financing technologies.

Coherence of workshop: Following a brief presentation of benefits and risks of DTHF from the perspective of the World Health Organisation (WHO), the workshop will present four case studies: (1) Rwanda, where a near universal coverage systems is supported by the digital Mutuelle Membership Management System (3MS) facilitating revenue collection through mobile money; (2) Ghana, where the National Health Insurance Scheme (NHIS) is using a Mobile Renewal system to increase coverage rates, and where an electronic claims management system has been recently introduced; (3) Kenya, where a wide range of digital innovations for revenue raising and purchasing were introduced in the private sector, which are now (partially) integrating the public coverage system; and (4) Madagascar, where experiences with private sector initiatives could support the development of national health insurance. Finally, we will outline the research agenda of the DigitalUHC consortium and collect input on salient topics for policy-makers and researchers.

**Format:**

The workshop is organised as a round-table panel discussion with panel members giving brief (5 min) introductory presentations before engaging in discussions with other panel members and the audience about cross-cutting issues, including (1) potential benefits of DTHF as well as risks and challenges; (2) the importance of politics and policies for reaping the benefits and minimizing the risks; and (3) the future research agenda. Several polling rounds will engage the audience and collect input on these topics, e.g. by asking participants about (a) examples of digital technologies that they know of, (b) their assessment of potential benefits, risks, and challenges, (c) necessary regulations in different countries, and (d) topics for future research.

**Funding:**

German Alliance for Global Health Research (GLOHRA) with funds from the Federal Ministry of Education and Research (BMBF).

**Key messages:**

• Digital technologies for health financing may help countries to achieve universal health coverage.

• Politics and policies are important to reap the benefits and minimize potential risks.

**Speakers/Panellists:**

**Inke Mathauer**

WHO, Geneva, Switzerland

**Regis Hitimana**

Rwanda Social Security Board, Kigali, Rwanda

**Daniel Opoku**

KNUST, Kumasi, Ghana

**Diana Ratsiambakaina**

Ministry of Health, Antanarivo, Madagascar

**Abdhalan Ziraba**

APHRC, Nairobi, Kenya

